# Assessment of the heterogeneous impacts of global value chain participation on Sustainable economic growth and environmental quality

**DOI:** 10.1016/j.heliyon.2024.e35348

**Published:** 2024-07-31

**Authors:** Amar Razzaq, Pomi Shahbaz, Shamsheer ul Haq, Yewang Zhou, Sahar Erfanian, Azhar Abbas

**Affiliations:** a Business School, Huanggang Normal University, No. 146, Xinggang 2nd Road, City Development Zone, Huanggang, 438000, China; b Department of Economics, Division of Management and Administrative Science, University of Education, Lahore, 54770, Pakistan; c Institute of Agricultural and Resource Economics, University of Agriculture, Faisalabad, 38040, Pakistan

**Keywords:** Global value chain, Carbon footprints, Sustainable economic growth, Environmental sustainability

## Abstract

The Global Value Chain (GVC) is an essential aspect of sustainable economic growth and environmental quality in countries that participate in it. Therefore, comprehending the relationship between GVC and economic growth and carbon emissions is critical to achieving global climate neutrality targets. However, there is a paucity of knowledge regarding the impacts of disaggregated levels of GVC on economic growth and carbon emissions in countries with different income levels. In this study, we utilized the SYS-GMM model to explore the relationships between backward and forward GVCs and the economic growth and carbon emissions of 42 lower-middle, 36 middle-upper, and 48 high-income countries using data from 1995 to 2018. Our key findings suggest that forward GVC significantly increases economic growth and reduces CO_2_ emissions. Conversely, backward GVC reduces the economic growth and CO_2_ emissions of high-income countries. We also found that upper-middle-income economies can enhance their economic growth and reduce CO_2_ emissions by increasing their backward GVC. In contrast, lower-middle-income economies can increase their economic growth by participating in both forward and backward GVCs. However, higher levels of participation in both GVC components result in increased CO_2_ emissions. Our findings emphasize the importance of considering income levels when analysing the impact of GVC participation on economic growth and environmental sustainability. Overall, the relationship between economic growth and CO_2_ emissions with backward and forward GVCs varies significantly across country categories.

## 1 Introduction

Climate change is the most significant threat to sustainable economic development, and greenhouse gases are considered the main cause of global climate change. So, reducing greenhouse gases is the key to reducing the effects of climate change on sustainable economic growth. However, since the beginning of the 21st century, CO_2_ emissions, which make up about two-thirds of all greenhouse gas emissions, have been continuously increasing [1]. Moreover, international trade has also expanded a lot during the same period, which has had an impact both on CO_2_ emissions and global economic growth. Thus, the nexus of economic growth, CO_2_ emissions, and trade has become an important topic for environmental sustainability and economic development [2]. These interactions are made more difficult by the development of global value chains (GVCs), which are the outcome of vertical specialization and international inter-industry trade.

Globalization has profoundly transformed the landscape of international trade and production, leading to the emergence of Global Value Chains (GVCs) as a dominant feature of the global economy. GVCs involve the fragmentation of production processes across countries, allowing each participant to specialize in specific stages of the value chain. This intricate network of cross-border activities has significantly contributed to economic growth and development, facilitating increased global integration and resource allocation efficiency [3,4]. The primary topics of studies on the link between GVCs and CO_2_ emissions are CO_2_ emissions along GVCs and the effect of GVC participation on CO_2_ emissions. According to Meng et al. [5], CO_2_ emissions along the production linkages of GVCs account for a significant portion of total carbon emissions at the global level.

The global economy has transitioned into the (GVC) era as intra-industry trade takes on a more significant role in international trade [6]. The global distribution of the production processes is a feature of GVC [7]. With GVC, a nation does not completely produce a product but only participates in a specific task or process. GVC trade is seen as a relatively simple path to industrialization and has changed the global structure of production [8]. GVC gives nations access to competitive markets and innovative and advanced technologies and assists nations in making their markets more competitive [9]. GVC combines both developed and developing nations to contribute to the production process. Grossman and Helpman [10] argue that technology enhances economic growth in the long term. GVC trade entails importing from and exporting to more than two nations. An economy needs both importing and exporting because importing boosts the effectiveness of capital accumulation, technological diffusion, and local innovation [11]. Thus, GVCs promote cross-border knowledge, skills, investments, and changes in human capital, in addition to international trade in products and services [12].

Three strands of literary works may be found on this topic. The first set of studies [6,13,14] supports a positive relationship between trade openness and CO_2_ emissions. This demonstrates that participation in GVCs may stop environmental damage and conserve energy. First, “technology spillovers” and “labor transfer” that result from participation in GVCs aid in the transmission of environmental and new energy technologies [15,16]. Second, taking part in GVCs helps spread technology and share technical knowledge while cutting down on emissions [17,18]. The second body of research backs up a negative link between global trade and CO2 emissions [[19], [20], [21]]. First, taking part in GVCs is linked to longer distances between network nodes, and when distances are longer, CO_2_ emissions from trucks are higher [22]. Second, involvement in GVCs, where greater backward links may lead to higher energy usage, drives the rise of the global energy footprint [23]. According to the third group of studies, the relationship between international trade participation and CO_2_ emissions may vary or be neutral across emission levels [[24], [25], [26]].

Therefore, the mechanism linking GVC, economic growth, and environmental impacts is intricate and involves several factors. GVC enable specialization, leading to economic growth and higher income levels. However, industrialization and trade-related activities may increase CO_2_ emissions. Import/export-related emissions and sectoral composition also play important roles. Technology transfer can reduce emission intensity, while stricter environmental regulations and sustainable development goals promote greener practices. Adopting cleaner technologies and integrating environmental considerations can mitigate CO_2_ emissions and foster sustainable economic growth through GVC participation.

Lower-middle, upper-middle, and high-income countries face diverse economic and environmental challenges owing to varying levels of industrialization, natural resource endowments, and policy frameworks [[27], [28], [29], [30]]. Forward and backward participation in GVCs play a crucial role in shaping the economic trajectories of countries across different income groups. Country participation allows them to benefit from expanding global markets and take advantage of their comparative advantages in specific industries or sectors [31,32]. Similarly, they can integrate into global supply chains and access advanced technologies and expertise [33]. Moreover, the impact of GVC participation varies across income groups because of their distinct economic characteristics and developmental stages. Lower-middle-income countries participate in GVC to boost their export competitiveness and attract foreign direct investment, leading to job creation and industrial development [34]. Conversely, upper-middle-income countries strengthen their position in global production networks and move up the value chain by increasing the sophistication of their production processes [35]. High-income countries may engage in GVC participation to maintain their global competitiveness in advanced industries and sustain economic growth [36]. Thus, understanding how forward and backward GVC participation interact with economic growth and environmental quality in these countries is essential for designing tailored strategies for sustainable development.

CO_2_ emissions but failed to consider the following aspects. First, prior studies on the linkage of GVCs ignored the income differences among countries while looking at the relationship of GVCs with CO_2_ emissions and economic growth. The association between GVCs with economic growth and CO_2_ is expected to be different for different income countries due to technology disparities. GVC trade consists of forward participation (FP) and backward participation (BP) components. Second, previous studies on this topic did not differentiate between BP and FP while looking at the GVCs with economic growth and CO_2_ emissions. Against the above background, this study makes the following contributions to the prior literature in the area: First, it represents an early attempt to analyze the impact of both backward and forward GVCs on CO_2_ with respect to the income categorization of the countries. Second, this study analyzes the relationships of backward and forward GVCs with economic growth based on income levels in the country.

The findings of this study will provide valuable insights for policymakers and stakeholders in designing strategies that maximize the benefits of GVC participation while mitigating potential risks. For lower-middle-income countries, this study offers guidance on leveraging forward or backward participation to drive sustainable economic growth and foster inclusive development. For upper-middle-income countries, understanding the impact of forward or backward participation will help to identify opportunities to upgrade production processes and enhance competitiveness. High-income countries can benefit from a comprehensive analysis of both forward and backward participation to maintain their position in global value chains and foster innovation in advanced industries. Therefore, the main objective of the current study is to explore the effects of F-GVC and B-GVC along with some other variables on economic growth as well as an environmental indicator (CO_2_ emissions) by considering the income disparities among nations.

The remainder of this article is organized as follows: Section 2 presents the relevant prior literature. Section 3 discusses the research approach, explains the study materials, and presents the econometric approach utilized in the study. Section 4 presents the model estimation results and discussion. Finally, section 5 concludes with policy implications.

## 2 Literature review

“GVCs” stand for Global Value Chains. Global Value Chains are a significant aspect of the modern global economy, where different stages of production of goods and services are spread across various countries. Instead of a single company producing a finished product entirely in one location, GVCs integrate different companies, industries, and countries to create a final product or service [37]. Thus, GVCs, as defined by Gibbon et al. [38], represent interconnected relationships between firms and other entities, enabling the geographical and organizational restructuring of economic production. Companies often pursue global efficiency by strategically assigning distinct production activities to foreign locations [39].

The components of GVCs include input suppliers, producers/manufacturers, distributors/wholesalers/retailers, marketing and advertising, support services, and end users. Input Suppliers describe the provision of raw materials, intermediate goods, components, and services required for the production process. Input suppliers may be domestic or located in other countries. Producers/manufacturers include firms that are responsible for the physical transformation of inputs into intermediate or final goods. Manufacturers may assemble, process, or refine the inputs received from various suppliers. Distributors/wholesalers/retailers describe the process of distributing goods once they are produced, and they must be distributed to end consumers or retailers. Distributors play a crucial role in moving products from the manufacturers to the retail market. Marketing and Advertising include promotional activity. This component involves promoting and advertising products to increase consumer demand. Marketing strategies can vary across countries to cater to cultural and regional preferences. Next is Research and Development (R&D): Companies may distribute R&D activities across different locations to take advantage of specialized skills and resources, leading to innovation and improved product development. The support services component of the GVC includes logistics, transportation, information technology, and legal, financial, and other services necessary to facilitate smooth operations along the value chain. End Consumers are the last component of the GVC. Timmer et al. [40] comprehensively described the components of GVC with real examples. They also focused on all these components in their particular context.

The growing importance of GVCs in global trade attracted international researchers who explored how GVCs contribute to economic growth and CO_2_ emissions. The growing popularity, complexity, and GVC participation have made it hard to figure out “who produces what for whom” [41] and the costs and benefits of participating in GVCs. This makes it hard to make policies that allow the government and businesses to take advantage of GVCs and reduce their negative effects, especially GHG emissions and pollution [5].

GVCs' participation is vital for knowledge spillover, and developing countries can greatly benefit from the knowledge available in GVCs to help them achieve high levels of innovation [18]. Moreover, the GVC participation of developing countries creates the opportunity for direct entry into global value trade without developing their value chain [42,43]. Mao [44] found a non-linear u-shaped relationship between GVC and economic growth and reported that the increase in GVC participation of latecomers contributes to economic growth.

Jangam and Rath [2] found the positive impact of GVCs as well as forward and backward linkages on economic growth and described the positive impact of sectoral GVCs on trade and economic growth. Similarly, Wu et al. [45] have described how the GVCs reconstruction caused by the China-USA decoupling affects the economy of China, and it can badly affect the country's GDP and employment. Hermida et al. [46] linked the high growth rate of GDP per capita of countries with their increased participation in GVCs. Furthermore, they stated that GVC participation and fragmentation are more important than gross export as a percentage of GDP. Fagerberg et al. [47] explored the role of GVCs in the economic growth of low-income countries. According to their findings, the increase in GVCs' participation limits growing capacity of low-income countries.

As global warming becomes a pressing issue, countries are urged to adopt individual and collaborative approaches to mitigate its impact. Climate change has increased international and domestic awareness, fostering efforts to identify ways to address this growing trend [48]. Awosusi et al. [49] used panel quantile regression to explore the impact of political risk, globalization, and technical innovation on the ecological footprint in BRICS economies. They found that economic expansion, non-renewable energy, political risk, and technical innovation increase the ecological footprint, whereas globalization decreases it. Adebayo et al. [50] studied the role of economic growth, energy use, and urbanization in environmental pollution in Latin American countries. They found that energy use and economic growth predicted CO_2_ emissions in Latin America. Adebayo et al. [51] examined South Africa's economic growth and CO_2_ emissions from 1980 to 2017, revealing a strong relationship between economic growth, renewable energy consumption, and CO_2_ emissions. Awosusi et al. [52] focused on economic growth in top energy transition economies and found that it leads to higher CO_2_ emissions. Additionally, Su et al. [53] demonstrated that positive income variation triggers CO_2_ emissions, whereas negative income variation has a neutral effect. They also observed that a negative change in trade openness increases CO_2_ emissions, whereas a positive change mitigates CO_2_ emissions.

Apart from contributing to economic growth, GVC participation also has environmental implications in terms of carbon foot prints. Wang et al. [6] derived the U-shaped relationship between GVCs and CO_2_ emissions. They also stated that the GVC participation effects of worsening CO_2_ emissions can be lowered by increasing R&D investment. Zhu et al. [54] explored a link between GVC participation and CO_2_ emissions spatially. Their findings indicated strong spillover effects in the country as well as in neighboring countries. They also said that the manufacturing sector was bigger than the services sector, and that high-tech manufacturing sub-industries were bigger than low-tech manufacturing sub-industries, which meant that GVC participation had strong effects on CO_2_ emissions through spillover. Meng et al. [5] determined the emission of CO_2_ systematically at the sector, country, and bilateral level and described who produces the value and who produces the emission form, which is also a critical point to trace in GVC linkages. Qian et al. [55] explored a link between GVCs and CO_2_ emissions in the countries of the Regional Comprehensive Economic Partnership. They determined that forward participation reduces their emissions while backward participation increases their emissions. They described a negative association between forward GVC participation and CO_2_ emissions. They further delineated a positive association between backward GVC participation and CO_2_ in these countries.

The existing literature on the impact of GVC and economic growth can be categorized into three distinct groups:1) studies that consider the impact of GVC on economic growth only (e.g. Refs. [2,[45], [46], [47]]) but do not focus on the environment. 2) Studies considering the implications of GVC on the environment, ignoring economic growth (e.g. Refs. [6,54,55]). 3) Studies examining the impact of forward and backward GVC on economic growth [2,56], without considering their environmental implications. Moreover, the literature lacks segregation of countries based on their potential or income when studying the impact of GVC on economic growth and the environment in one place. Therefore, the current study aims to address this research gap by considering both forward and backward GVC participation in relation to economic growth and the environment in lower-, middle-, and high-income countries.

### 2.1 Theoretical aspect of GVC, economic growth, and the environment

The theoretical model explaining the link between GVCs, economic growth, and environmental pollution is the Pollution Haven Hypothesis (PHH) [57,58]. This suggests that multinational corporations move production to countries with weaker environmental regulations to reduce compliance costs, leading to increased economic growth and job creation [59]. Justifications for PHH include regulatory arbitrage, resource-intensive industries' attraction to countries with lax environmental standards, and potential innovation spurred by stricter regulations [[60], [61], [62]]. GVCs based on comparative advantage can concentrate industries in specific countries, thereby impacting the environment [63,64]. Participation in GVCs can boost exports, foreign investment, and economic growth [65,66]. However, the pollution haven effect can lead to “pollution leakage” without reducing the global pollution levels [67].

The theoretical link between GVCs, economic growth, and environmental pollution is a complex interplay between various economic and environmental factors. Based on the above theoretical justification, this link between GVC, economic growth, and the environment can be explained as follows. GVCs allow countries to specialize in specific stages of the production process, where they have a comparative advantage. This specialization can lead to increased productivity, efficiency, and competitiveness, fostering economic growth. GVCs often involve foreign direct investment, as multinational corporations establish operations in different countries to access resources, labor, or markets. FDI can lead to technology transfer, infrastructure development, and job creation, thus positively impacting economic growth [68]. Because GVCs facilitate trade and production, they can lead to increased income levels for the countries involved [69]. Higher income can result in increased consumption and demand for goods and services, further driving economic growth. GVCs create job opportunities in countries participating in different stages of the value chain [36], which can reduce unemployment rates and contribute to overall economic development. GVCs can foster the transfer of technology, skills, and know-how from advanced economies to developing countries [70]. Access to new technologies can boost productivity and innovation and promote economic growth. Countries in GVCs may have varying levels of environmental regulation. Firms may choose to locate certain production stages in countries with weaker environmental standards in order to reduce compliance costs. This can lead to higher environmental pollution in these regions, a phenomenon known as the Pollution Haven Hypothesis. Resource Extraction and Environmental Impact: GVCs often involve resource extraction and production activities that can have significant environmental impacts [3] such as greenhouse gas emissions. GVCs rely on the transportation of goods over long distances, which can lead to additional greenhouse gas emissions and air pollution [71]. Stricter environmental regulations in certain countries may incentivize firms to adopt cleaner technologies and practices and promote sustainable production and innovation [72,73]. The environmental consequences of GVCs can extend beyond national borders through international trade and production networks, thereby impacting the global environment.

## 3 Materials and methods

### 3.1 Measuring the forward and backward GVCs

The nature of the trade has changed in the last few decades. The major change that is linked to the production process has witnessed the segmentation of the production process across the countries, which has started a new pattern of trade in the world. Now, firms in many different countries want to join the complex production networks. They do this by working with both domestic and international firms to add different inputs to their goods and services [74]. With the growing adoption of the GVCs around the world, its two types of trade linkages, such as “Forward GVCs (F-GVC)" and “Backward GVCs (B-GVC)” have their own practical and beneficial importance. The current focus of this study is on these two trade linkages, which are measured and named differently in the literature. Ndubuisi and Owusu [33] described participation in GVC as “buyer” and described what is known as “Backward GVC Participation” (upstream links in a global production chain which describes that countries buying the foreign inputs that can further be used in the production of the goods they export), and participating as “seller” is called “Forward GVC participation” (downstream links in an international production chain which describes the exporting of domestically produced inputs to the third economies). Therefore, the backward GVC participation is calculated by considering the foreign value added that manifests the country's gross export. The mathematical form of B-GVC participation is given below (Eq. (1)):

1 $$B-GVC=\frac{{FVA}_{ct}}{{GE}_{ct}}$$

here, FVA is the foreign value added of a country c in a year t and GE is the gross export of a country c in a year t. A higher ratio of FVA and GE means high B-GVC participation. The F-GVC participation is measured by using the following Eq. (2):

2 $$F-GVC=\frac{{DVX}_{ct}}{{GE}_{ct}}$$

here, DVX is the domestic value added of a country c in a year t and GE is the gross export of a country c in a year t. A higher ratio of DVX and GE in a country means greater F-GVC participation. Similarly [75], have considered these two international linkages with the term “Vertical Specialization (VS)", describing the VS concerning import (buyer) as B-GVC and the VS concerning export (seller) as F-GVC. To carry out our analysis, we have used the Eora-MRIO (Multi-Regional Input-Output) data set.

Disaggregating GVCs into backward and forward participation is important because it provides a more detailed and nuanced understanding of how different countries and industries are integrated into the global production network. Backward and forward participation refer to the direction of the value chain in which a country or industry is involved, and the significance of disaggregating GVCs into backward and forward participation lies in the following aspects: i) Disaggregation assists in identifying comparative advantages. Understanding which countries or industries engage in backward or forward participation can help identify their specific comparative advantages. This knowledge is vital for optimizing efficiency and for making informed policy decisions regarding trade, investment, and industrial development. Second, ii) Disaggregating GVCs allows us to analyze how value addition is distributed across countries and regions. It helps identify which countries capture a larger share of value through more advanced stages of production and which countries may have limited opportunities for value capture. Third, iii) governments and policymakers can use this information to formulate targeted policies to support industries in moving up the value chain. It enables them to focus on developing specific sectors, enhancing skills, technology transfer, and improving infrastructure to foster forward participation and create more value-added activities; and iv) Disaggregating GVCs helps in understanding the vulnerabilities and risks associated with supply chains. Countries heavily involved in backward participation may face disruptions in their supply of inputs, affecting their own production capabilities and the stability of the global value chain; and v) recognizing the roles of countries in different segments of the value chain can lead to enhanced trade and investment opportunities. Countries with complementary roles can foster partnerships and collaborations by benefiting from each other's strengths. Therefore, disaggregating GVCs into backward and forward participation is crucial for understanding the intricacies of global production networks, optimizing economic activities, and formulating effective policies for economic development and resilience [[76], [77], [78], [79], [80]].

Disaggregating Global Value Chains (GVCs) into backward and forward participation is crucial when considering the environment for several reasons: i) it allows for accurate environmental impact assessments, identifying hotspots and areas for improvement in the value chain; ii) companies can trace the origins of raw materials, promoting sustainable sourcing practices; iii) targeted efforts can be made to green different stages of the value chain; iv) governments can implement more effective environmental regulations based on specific GVC activities; v) understanding emissions at different production stages aids in climate change mitigation strategies; vi) circular economy initiatives can be implemented by identifying recycling opportunities; vii) assessing vulnerabilities and building resilience to environmental disruptions becomes possible. Disaggregating GVCs with a focus on the environment promotes sustainability, addresses environmental challenges, and ensures a resilient and eco-friendly global-production network.

The data on GDP per capita was taken from the World Bank's World Development Indicators to capture the impact of F-GVC and B-GVC on the economic development of countries. The GDP per capita is the main variable that is widely used as a mirror of social welfare [81], as a reflection of economic development [74,82]. Similarly, to capture the impact of F-GVC and B-GVC on the environment, we considered the CO_2_ emissions of countries. CO_2_ emissions are one of the most important elements being highly focused on by the research community when they consider the environment. CO_2_ emissions are a major cause of climate change and have a harmful and irreversible effect on the economy [83]. Moreover, GVC participation and emissions are also strongly linked. Zhang et al. [84] stated that GVC participation has reshaped the CO_2_ emissions of countries.

The multi-country analysis also considered a few additional baseline explanatory variables. We used the population of countries and governess effectiveness to control the endogeneity of predictors. The foreign direct investment (FDI) also plays an important role in economic growth, which also facilitates trade, advanced capital inflow, and competitive market share of global standards [85]. Therefore, FDI data from the World Bank's World Development Indicators was also considered as an explanatory variable in the current study. Moreover, the human capital development index and Index of Economic Freedom were also used as model explanatory variables in the study. The data from Penn World Table 9.0's human capital development index demonstrate the importance of a skilled and more literate labor force. The Heritage Foundation's Index of Economic Freedom shows how freedom and free markets affect different parts of the world. The key variables of the study and their sources are listed in Table 1.

**Table 1:** Variables of the study and their sources.

Variables	Source	Links
F-GVC & B-GVC	Eora-MRIO	https://worldmrio.com/unctadgvc/
GDP per Capita	WDI	https://databank.worldbank.org/source/world-development-indicators
CO_2_ Emission	WDI	
FDI	WDI	
Human Capital Index	Penn World Table 9.0	https://www.rug.nl/ggdc/productivity/pwt/pwt-releases/pwt9.0?lang=en
Economic Freedom Index	Heritage Foundation Index of Economic Freedom	https://www.heritage.org/index/

### 3.2 Categorization of countries

Based on the per capita GNI, the World Bank divides countries into low-, lower middle-, upper middle-, and high-income countries. Countries are classified as low-income if their GNP per capita is less than or equal to $1045; lower-middle income if their GNP per capita is between $1046 and $4095; upper-middle income if their GNP per capita is between $4096 and $12695; and high-income if their GNP per capita is greater than or equal to $12696 [86]. Afterward, during the development of panels for these four categories, the study had to leave out low-income countries due to the unavailability of relevant data. Thus, the current study focused on the other three categories, which included 42 lower-middle-, 36 upper-middle-, and 48 high-income economies. List of sampled countries is provided in the appendix A1.

### 3.3 Aggregate correlation linear relationship

**Fig. 1:**
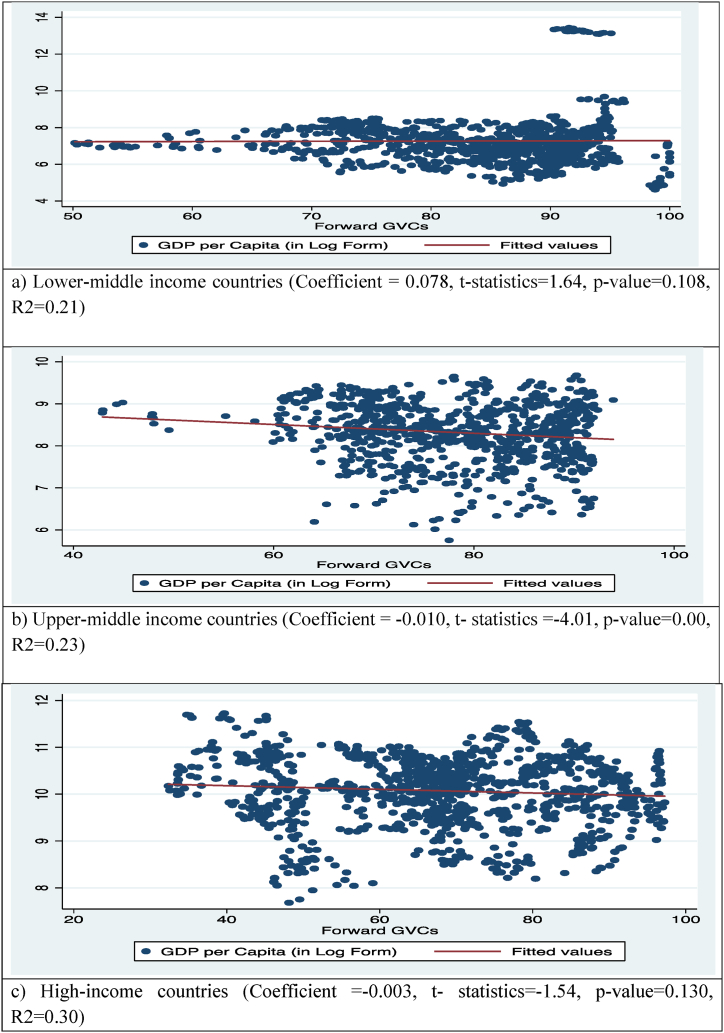
GDP per capita and F-GVC participation of countries.

**Fig. 2:**
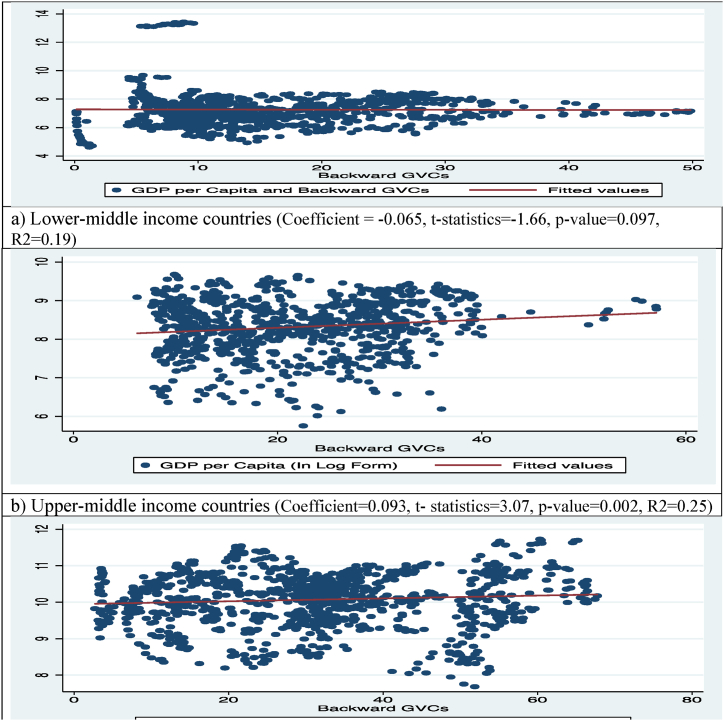
GDP per capita and B-GVC participation of countries.

**Fig. 3:**
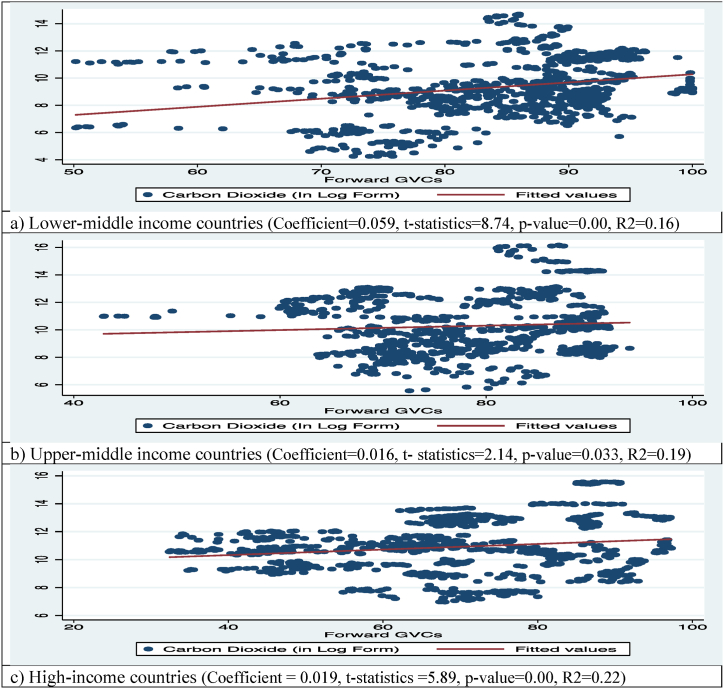
CO_2_ emission and F-GVC participation of countries.

**Fig. 4:**
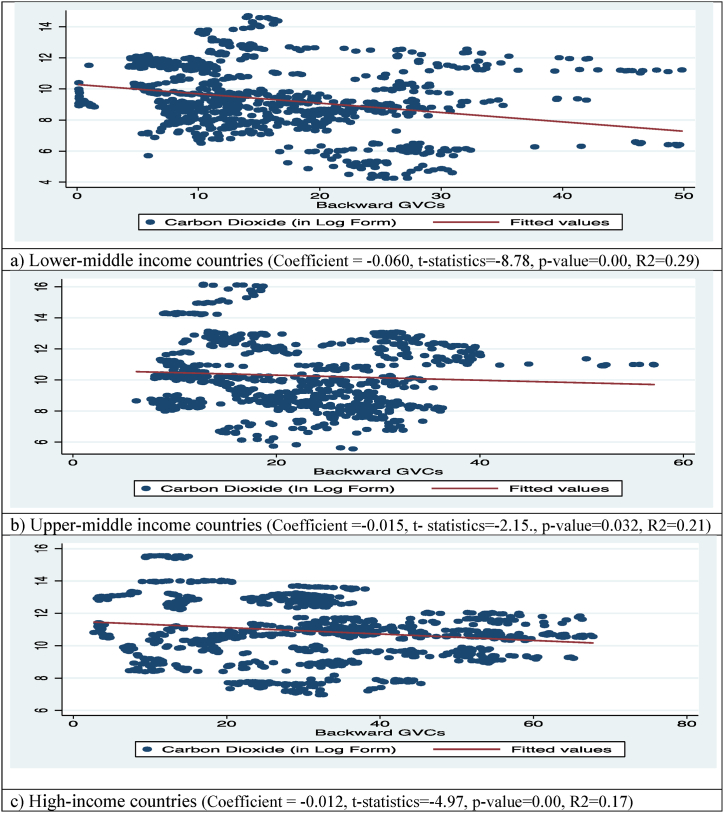
CO_2_ emission and B-GVC participation of countries.

The growing participation of the countries in the GVC, either forward or backward, may have multidimensional impacts on the economy as well as the environment. The F-GVC and B-GVC participation of the three categories of countries described above may have different impacts on their economies and the environment. Fig. 1, Fig. 2, Fig. 3, Fig. 4 depicts the F-GVC and B-GVC trends in relation to GDP per capita and CO_2_ emissions.

Fig. 1 presents the aggregate relationships between the F-GVC and GDP per capita of all three categories of the countries. Fig. 1-a depicts the aggregate association between F-GVC and GDP per capita of lower-middle income economies. A positive relationship was found between F-GVC and the GDP/capita of lower upper-income countries. It shows that the one-unit rise in F-GVC participation will increase the GDP/capita by 0.078 % points. In the case of upper-middle-income countries (Fig. 1-b), the aggregate connection indicates a negative association between the F-GVC and GDP/capita, which means that each additional point increase in the F-GVC causes a 0.010 % point decrease in GDP/capita. The aggregate relationship between F-GVC and GDP per capita in high-income countries was found to be negative (Fig. 1-c), indicating that every additional unit increase in F-GVC is linked with a 0.003 % point decrease in GDP/capita.

Fig. 2 shows the aggregate association between B-GVC and GDP/capita in all three categories of the countries. Fig. 2-a shows the negative aggregate relationship between B-GVC and GDP/capita of lower-middle income economies, which means that one unit rise in B-GVC is related with a 0.065 % point decrease in GDP per capita of lower-middle income countries. The positive aggregate link between B-GVC and GDP/capita of upper-middle-income economies in Fig. 2-b explains the 0.093 percentage point rise in GDP per capita with each additional unit of B-GVC. Fig. 2-c shows that the B-GVC and GDP per capita have a positive overall relationship. This means that a one unit rise in B-GVC leads to a 0.0028 % point increase in GDP per capita in countries with higher income nations.

The lower-middle income countries' overall relationship between F-GVC and CO_2_ emissions is shown in Fig. 3. Fig. 3-a depicts the aggregate relationship between F-GVC and CO_2_ emissions in lower-middle income countries. The aggregate link between F-GVC and CO_2_ emissions was positive and 0.059 % point increase in CO_2_ emissions is associated with an additional unit rise in F-GVC in these countries. In case of upper-middle income countries (Fig. 3-b), there is a significant positive aggregate relationship between their F-GVC participation and CO_2_ emissions. These countries' additional one unit of F-GVC results in a 0.016 percentage points increase in CO_2_ emissions. A similar positive and significant aggregate relationship between F-GVC and CO_2_ emissions of higher countries was also found. This shows that a one-unit increase in F-GVC of these countries causes a 0.019 % point increase in CO_2_ emissions (Fig. 3-c).

The aggregate relationship between B-GVC and CO_2_ emissions for all country categories is shown in Fig. 4. Fig. 4-a shows that one additional unit increase in B-GVC of lower-middle income countries causes a 0.060 % point decrease in CO_2_ emissions. Similarly, a negative and significant aggregate relationship was noted between B-GVC and CO_2_ emissions in upper-middle (Fig. 4-b) and high-income (Fig. 4-c) countries. This overall relationship shows that one more unit of B-GVC participation causes CO_2_ emissions to drop by 0.015 and 0.012 % points in upper-middle- and high-income countries, respectively. The patterns of aggregate relationships of F-GVC and B-GVC with GDP per capita and CO_2_ emissions in the above figures describe that F-GVC and B-GVC participation are important drivers of environmentally friendly economic growth for all three country categories.

### 3.4 Empirical model construction

The current study used panel regression and SYS-GMM to account for the endogeneity concerns in the GDP/capita and CO_2_ emissions. The endogeneity problem that motivates the choice of system-GMM estimation arises when the explanatory variables in a regression model are correlated with the error term. This correlation violates one of the key assumptions of the Ordinary Least Squares (OLS) regression, leading to biased and inconsistent parameter estimates. The endogeneity problem often occurs in various economic and social science studies, especially when dealing with dynamic panel data models and simultaneous equation models. In these cases, the error term may be correlated with the past or future values of the explanatory variables, creating endogeneity [87]. Thus, the utilization of System GMM is highly recommended because of its demonstrated effectiveness in mitigating bias arising from measurement errors, unobserved heterogeneity, variable omission, and the prevalent issue of endogeneity that often impacts the dependent variable [88,89].

Da Silva and Cergueira [90] described that the system-GMM provide highly precise with minimum biased results when N dimension in the panel is small. Similarly, the system-GMM provides the efficient and consistent parameter estimates when independent variables are not strictly exogeneous. System GMM also overcome the problem of autocorrelation and heteroskedasticity [[91], [92], [93]]. Two diagnostic tests like Sargan test of over-identification and Arellano-Bond (AR2) autocorrelation test also confirmed the consistency of system-GMM estimation. AR2 test confirmed the absence of autocorrelation in the models, and Sargan tests ensured the validity of instruments used in each model. Moreover, the non-overlapping range of data (N > T) endorse the use of system GMM in the current study. For robustness, in addition to foxed effect (FE) model, we have applied the panel corrected standard error (PCSE) is used to confirm the results’ robustness, which also eliminate the issue of cross section dependence in the panel.

A separate SYS-GMM model was executed for each category of the economy to estimate the possible effect of the predictors on economic indicators (GDP per capita) as well as environmental indicators (CO_2_ emissions). The general form of the model is as below, as specified by Ref. [94]:

For Economic Growth;

3 $${GDP}_{cit}=\beta_{o}+\beta_{1}{F-GVC}_{cit}+\beta_{2}{B-GVC}_{cit}+\beta_{3}{FDI}_{cit}+\beta_{4}{HC}_{cit}+\beta_{5}{EF}_{cit}+\varepsilon_{it}$$

For Environmental indicator;

4 $${{CO}_{2}}_{cit}=\beta_{o}+\beta_{1}{F-GVC}_{cit}+\beta_{2}{B-GVC}_{cit}+\beta_{3}{FDI}_{cit}+\beta_{4}{HC}_{cit}+\beta_{5}{EF}_{cit}+\varepsilon_{it}$$

where ${GDP}_{cit}$ is GDP per capita, ${{CO}_{2}}_{cit}$ is quantity of CO_2_ emission, ${F-GVC}_{cit}$ is forward GVC, ${B-GVC}_{cit}$ is backward GVC, ${FDI}_{cit}$ is foreign direct investment, ${HC}_{cit}$ is human capital development index and ${EF}_{cit}$ is economic freedom of country i at time t from country panel c, and ε_it_ is an error term. Some econometric issues, like the endogeneity problem regarding the empirical assessment of the effects of F-GVC and B-GVC with other explanatory variables on GDP/capita and CO_2_ emissions, can be solved with the below dynamic equation for both dependent variables. For economic growth:

5 $${GDP}_{cit}-{GDP}_{cit-1}=\beta_{o}+\alpha_{o}{GDP}_{cit-1}+\beta_{1}{F-GVC}_{cit}+\beta_{2}{B-GVC}_{cit}+\beta_{3}{FDI}_{cit}+\beta_{4}{HC}_{cit}+\beta_{5}{EF}_{cit}+\mu_{t}+\eta_{t}+\varepsilon_{it}$$

6 $$\Delta {GDP}_{cit}=\alpha_{o}{GDP}_{cit-1}+\theta X_{it}+\mu_{t}+\eta_{t}+\varepsilon_{it}$$

For environmental indicator

7 $${{CO}_{2}}_{cit}-{{CO}_{2}}_{cit-1}=\beta_{o}+\alpha_{o}{{CO}_{2}}_{cit-1}+\beta_{1}{F-GVC}_{cit}+\beta_{2}{B-GVC}_{cit}+\beta_{3}{FDI}_{cit}+\beta_{4}{HC}_{cit}+\beta_{5}{EF}_{cit}+\mu_{t}+\eta_{t}+\varepsilon_{it}$$

8 $$\Delta {{CO}_{2}}_{cit}=\alpha_{o}{{CO}_{2}}_{cit-1}+\theta X_{it}+\mu_{t}+\eta_{t}+\varepsilon_{it}$$

where $\Delta {GDP}_{cit}$ and $\Delta {{CO}_{2}}_{cit}$ is the change in GDP and CO_2_ in country i at time t from country panel c. The term $\mu_{t}\;and\;\eta_{t}$ depicts all shared elements that impact the economies and detect unobserved country-effect features. $X_{it}$ is equal to ${F-GVC}_{cit},{BGVC}_{cit},{FDI}_{cit},{HC}_{cit},and\;{EF}_{cit}$, and θ = (***β***_0_, ***β***_1_, ***β***_2_, ***β***_3_, …., ***β***_5_).

The dynamic equations described above have been used with panel data, which can deal with the endogeneity problem with explanatory variables. This endogeneity problem affects the causality of regressors such as F-GVC and B-GVC, foreign direct investment, human capital development, and economic freedom. Also, it can be said that the regressors are affected by the other endogenous variables in the different economies, and that they may cause the GDP and CO_2_ (which are called “dependent variables”) to change. As a result, in the presence of the endogeneity problem, ordinary least squares (OLS) regression may produce unreliable and biased results. Because of these issues, Arellano and Bond's [89] generalized method of moments (GMM) panel data estimation strategies were used in this study.

## 4 Results and discussion

The prime objective of the current research is to explore the effects of F-GVC and B-GVC along with some other variables on economic growth as well as an environmental indicator (CO_2_ emissions). The noteworthy point in Table 2 is that all the variables correlate with the GDP per capita (economic growth) and CO_2_ emissions. The F-GVC has a positive correlation with GDP for lower-middle economies and a negative correlation with GDP in the case of both upper-middle and high-income countries. In the case of lower middle economies, B-GVC has a negative correlation with GDP, while in the case of upper and middle economies; it has a positive correlation with GDP.

**Table 2:** Correlation structure of variables for lower-middle, upper-middle and high-income economies.

	1	2	3	4	5	6	7
**Lower-middle Economies**							
GDP per Capita	1.000						
Carbon Dioxide	−0.048	1.000					
F-GVC	0.340	0.363	1.000				
B-GVC	−0.354	−0.324	−1.000	1.000			
Foreign Direct Investment	0.309	−0.801	0.001	0.000	1.000		
Human Capital Index	−0.249	0.277	−0.285	0.285	−0.087	1.000	
Economic Freedom	0.239	−0.271	−0.236	0.237	−0.024	0.110	1.000
**Upper-middle Economies**							
GDP per Capita	1.000						
Carbon Dioxide	0.060	1.000					
F-GVC	−0.317	0.301	1.000				
B-GVC	0.374	−0.352	−1.000	1.000			
Foreign Direct Investment	−0.148	−0.748	−0.069	0.070	1.000		
Human Capital Index	0.321	0.265	−0.152	0.152	−0.024	1.000	
Economic Freedom	0.277	−0.296	−0.170	0.170	0.043	0.165	1.000
**High-income Economies**							
GDP per Capita	1.000						
Carbon Dioxide	0.131	1.000					
F-GVC	−0.339	0.252	1.000				
B-GVC	0.343	−0.252	−1.000	1.000			
Foreign Direct Investment	0.175	0.230	0.016	−0.017	1.000		
Human Capital Index	0.356	0.256	−0.218	0.218	0.033	1.000	
Economic Freedom	0.260	−0.210	−0.266	0.267	−0.031	−0.045	1.000

The F-GVC has a positive association and the B-GVC has a negative association with CO_2_ emissions in the case of all economies, irrespective of country category. In the case of both lower-middle and upper-middle-income economies, FDI has the highest negative correlation.

FDI has the strongest negative correlation in both lower-middle-income and upper-middle-income economies. All of these positive and negative relationships between other variables and GDP and CO_2_ are also interesting and should be looked into further.

The empirical results derived from the SYS-GMM model are presented in Table 3. The GMM model's significance tests, such as the Sargan and Hansan tests, as well as the second-order correlation, were all confirmed for all country categories. Empirical econometric findings need to confirm the validity of components because the minor invalidity of instruments may have severe implications for coefficients of regression [95]. Sargan or Hensen test for testing the over-identification, and Arellano-Bond [AR2] for testing the autocorrelation were employed to ensure the reliability of the data sets in the study. Therefore, we checked the relevance of the instruments by reporting the p-value of Sargan-Hansan and the 2nd order correlation (AR2). Sargan's p-values are higher than 0.05, indicating that the null hypothesis of over identification restriction, such as all instruments being valid and accepted, is correct. It was confirmed by observing the p-value related to the Sargan test for all three economies, which was greater than the conventional p-value (0.05), thereby indicating that all instruments are valid [96]. The p-value of the 2nd order correlation (AR2) showed that there were no mistakes in the specifications because it showed that the null hypothesis was false.

**Table 3:** Results of GMM (SYS) and FE models.

	GDP Model		CO_2_ Model	
	GMM (SYS)	FE (Robust)	GMM(SYS)	FE (Robust)
**High Income Countries**				
Gross Domestic Product (GDP) L1.	−0.00002** (0.00001)	–	–	–
CO_2_ L1.	–	–	0.0041* (0.0007)	–
Forward GVCs	0.1406* (0.0432)	−0.0981 (0.0437)	−0.0831** (0.0368)	−0.0577 (0.0266)
Backward GVCs	−0.2341* (0.0694)	0.1956 (0.0574)	−0.0553** (0.0230)	−0.0495 (0.0262)
Foreign Direct Investment	−0.0003 (0.0003)	−0.0003 (0.0003)	−0.0004* (0.0001)	−0.0008 (0.0001)
Human Capital Index	0.8353** (0.3450)	0.7845 (0.2873)	1.1890 (0.8808)	0.1648 (0.8201)
Economic Freedom	0.0264** (0.0109)	0.0431 (0.0110)	−0.0690* (0.0073)	−0.0163 (0.0061)
Constant	25.40*** (13.55)	15.7119 (4.1858)	−5.8908 (9.6512)	7.0662 (3.2193)
R^2^	–	0.84	–	0.64
No. Of Obs.	1104	1152	1104	1152
Significance test (p-value)				
Sargan Test	8.12 (0.97)	–	35.69 (0.18)	–
Hansen Test	7.54 (0.98)	–	22.37 (0.21)	–
2nd order Correlation	−0.71 (0.48)	–	−0.94 (0.34)	–
No. Of Instruments	28		28	
**Upper Middle Income Countries**				
Gross Domestic Product (GDP) L1.	−0.00002 (0.00008)	–	–	–
CO_2_ L1.	–	–	0.0003** (0.0001)	–
Forward GVCs	−0.4261** (0.2043)	−0.7823 (0.2049)	3.1216 (3.4233)	1.0208 (2.4365)
Backward GVCs	0.5085** (0.2245)	0.5447 (0.2225)	−3.1251** (1.4190)	−3.0256 (1.3576)
Foreign Direct Investment	−6.3208 (5.0900)	−1.1779 (1.1871)	2.2346 (2.8541)	0.9584 (1.9870)
Human Capital Index	1.8343* (0.4488)	0.7413 (0.4071)	−6.1310*** (3.3464)	−4.9845 (2.9307)
Economic Freedom	0.0439* (0.0152)	0.0346 (0.0136)	−0.1439*** (0.0801)	−0.1731 (0.0782)
Constant	22.58** (10.9814)	36.81 (6.8343)	23.71 (24.4321)	37.25 (5.2223)
R^2^	–	0.86	–	0.56
No. Of Obs.	828	864	828	864
Significance test (p-value)				
Sargan Test	8.64 (0.96)	–	19.72 (0.34)	–
Hansen Test	10.16 (0.93)	–	3.29 (0.99)	–
2nd order Correlation	0.55 (0.58)	–	1.27 (0.21)	–
No. Of Instruments	21		21	
**Lower Middle Income Countries**				
Gross Domestic Product (GDP) L1.	−0.0007** (0.0003)	–	–	–
CO_2_ L1.	–	–	0.0008*** (0.0004)	–
Forward GVCs	0.0281 (0.1456)	−0.0732 (0.1580)	1.2588** (0.6319)	1.7410 (0.5965)
Backward GVCs	0.0316* (0.0100)	0.0653 (0.0184)	1.1733** (0.4645)	1.0063 (0.3735)
Foreign Direct Investment	0.7257* (0.2149)	0.4250 (0.1922)	1.4226*** (0.7606)	1.6431 (0.7214)
Human Capital Index	−0.4542 (0.9250)	−0.0735 (0.7979)	0.7185 (1.9933)	0.1069 (1.0985)
Economic Freedom	0.0256** (0.0123)	0.0243 (0.0097)	−0.0697 (0.1288)	−0.0986 (0.0986)
Constant	26.06 (28.1632)	9.039 (7.3796)	−14.8 (12.7212)	13.777 (6.1115)
R^2^	–	0.83	–	0.64
No. Of Obs.	966	1008	966	1008
Significance test (p-value)				
Sargan Test	1.16 (0.98)	–	9.13 (0.96)	–
Hansen Test	6.65 (0.99)	–	4.86 (0.99)	–
2nd order Correlation	−0.55 (0.58)	–	0.50 (0.62)	–
No. Of Instruments	32		32	

Note: The values presented in parenthesis are standard errors. FE (Fixed Effect) was used for robustness purpose. *, **, and *** denotes significance levels at 1 %, 5 %, and 10 % respectively.

There are a number of significant positive and negative coefficients in the results. In the case of high-income economies, we found a significant positive impact of F-GVC and a negative impact of B-GVC on the GDP per capita. In the case of upper-middle-income economies, we discovered a significant inverse relationship between F-GVC and a direct relationship between B-GVC and economic growth. In the case of lower-middle income economies, a significant positive impact of both F-GVC and B-GVC on economic growth (GDP per Capita) was found.

The significant positive impact of F-GVC and the negative impact of B-GVC on GDP per capita of high-income countries are due to their level of adoption of new technologies, as well as highly qualified human capital that is very expert in providing the highly valued domestic value-added for exports. For example, the automobile industry is one of the major examples of forward linkages that directly export to the rest of the world. In the USA, this industry majorly contributes to economic growth while absorbing 1.7 million people in the completion of all manufacturing procedures [97]. Similarly, Koopman et al. [98] described that countries having sophisticated technology and highly-skilled labor will have a greater share in forward GVC. For example, high-income countries such as the USA, Canada, Japan, Germany, Italy, etc. are more advanced in manufacturing high-technology-based goods for direct consumption such as automobiles and information and communication technologies (ICT), and their forward linkages in these goods have a high impact on economic growth and output growth (Schreyer, 2000). Moreover, Banga [99] found the forward GVC/linkages of the US, Japan, and UK is much stronger than their backward GVC/linkages, and China (an upper-middle income country) has strong backward GVC/linkages.

The positive impact of both F-GVC and B-GVC on the economic growth of lower-middle income economies can be explained by considering the lower-middle income economies’ participation in GVC as an opportunity for them to join the existing international supply chain instead of building one. As these countries are very poor in technology as well as have a low capacity to approach the international standard of trade, their cost of producing the goods and services for international trade may be high as compared to the other upper-middle and high-income economies. Therefore, the GVC, either forward or backward, provides the opportunity for lower-middle income economies to escape from the limitations imposed by the economies of scale, and they can easily rely on the production networks by making their connection with international firms. As a result, developing countries, such as lower-middle income economies, have begun to shift from producing final to intermediate goods, thereby shifting GVC upward and out of the assembly [100].

The countries that are specialized in sophisticated tasks and add more value to goods have more forward linkages. Similarly, the nations that concentrate on low value-added errands such as assembling have more backward linkages. Therefore, the backward/sourcing linkages are more valuable for the lower and upper middle-economies, while selling is a more attractive linkage for the high-income countries. Kummritz [100] described backward linkages in high-income countries as significantly contributing to their economies, whereas in lower and upper-middle income economies, the impact of backward linkages was positive and significant, whereas in high-income economies, this impact is negative. The textile and cotton industries in South-Asia countries such as India, Pakistan, and Bangladesh have major backward and forward linkages with other countries, which majorly contribute to the GDP per capita and the country's economic growth [101].

Our results also match with Lin and Wang [102], who described the impact of backward and forward linkages on the industrial up gradation of developed and developing countries. They concluded that the backward GVC has a stronger impact on the industrial up gradation of developing countries by adding value to imported intermediate inputs than the forward GVC, which contributes highly to the industrial up gradation of developed countries. In the same way, GVC participation allows developed countries outsource parts of tasks with low value-added and focus on tasks with high value-added [[103], [104], [105]]. Navas-Alemán [106] found that developing countries, including those with lower-middle and upper-middle incomes, can improve their economies by joining GVCs. For example, when developed countries give their technology to developing countries, their competitive advantages change. This permits the developing countries join the global production networks even though they do not have experts, or all the resources needed for production [[107], [108], [109], [110]]. In this way, developing countries can make more modern products by starting with simple tasks and then moving on to more complicated ones [101,102,111].

### 4.1 Other variables impact on economic growth

The results show that foreign direct investment (FDI) has a significant positive impact on the economic growth of lower-middle-income countries. FDI has many benefits, such as balancing out savings and investments and making it easier to use new technologies in making goods and services. Moreover, it increases tax revenue and human capital [112,113]. Similarly, it is one of the vital sources of the process of economic integration through the long-term benefits and connections among the countries. Thus, FDI has had a long-term positive impact on economic growth. In lower-middle income countries, they have the capacity to absorb technology and highly skilled labor. Moreover, FDI brings a sophisticated method of production as well as new technology with skilled labor, which contributes to their economic growth positively. Dinh et al. [114] also described the long-run positive impact of FDI on the economic growth of lower-middle-income economies.

Human capital development and economic freedom have a significant positive relationship with the economic growth of high- and upper-middle-income countries, while only economic freedom has a significant positive link with the economic growth of lower-middle-income countries. Our results are in line with De Hann and Sturm [115], Heckelman [116], and Doucouliagos and Ulubasoglu [117]. Human capital is measured with the human capital index, and it may contribute to economic growth by increasing the demand for highly skilled labor, scientists, and engineers. Pelinescu [118] and Neeliah and Seetanah [119] describe how human capital is an important determinant of economic growth.

### *4.2* *Impact of F-GVC and B-GVC on* CO_2_*emissions*

It is difficult to know whether the role of GVC in the context of the environment is favorable or not [120]. In the case of the high-income economies, a significant and negative impact of forward and backward GVC on CO_2_ emissions was found. In the case of the upper-middle income economies, the B-GVC has a significant negative impact on CO_2_ emissions. Both forward and backward GVC have a big negative effect on CO_2_ emissions in low- and middle-income countries. Because high-income countries have advanced technologies, they are more concerned about emissions than low-income countries. They can develop environmental protection policies and can ensure their application in the case of each economic activity. According to Lovely and Popp [121] and Nemati et al. [122], GVC participation can help countries protect their environment by developing and spreading environmentally friendly technologies. Moreover, the developed/high-income countries such as the UK, Japan, Germany, etc. can provide more sophisticated technologies or manufacturing intermediates to their downstream countries for further processing and assembling (Meng et al., 2018), which can also reduce their territory's CO_2_ emissions.

In general, Shi et al. [123] described that the increase in F-GVC would reduce carbon emissions more than that of B-GVC. But in the case of developed countries, they can reduce their carbon emissions by increasing their degree of F-GVC and B-GVC participation, while B-GVC, in the case of developing countries, can increase the carbon emissions. Qian et al. [55] explored the different spillover effects of F-GVC and B-GVC on CO_2_ emissions in RCEP countries. They stated that the increase in F-GVC reduces CO_2_ emissions while the increase in B-GVC increases CO_2_ emissions at the national level. Moreover, they explain that the increase in F-GVC participation in productive services industries and medium-to high-tech manufacturing can reduce CO_2_ emissions, while an increase in B-GVC participation of countries in low-tech manufacturing industries increases CO_2_ emissions. Therefore, the lower-middle income countries that have limitations on human capital and technologies in production and value addition are more reluctant to contribute to CO_2_ emissions as compared to the other upper-middle and high-income economies.

#### 4.2.1 Robustness checking

In addition of fixed effect model used as robustness purpose in Table 3, the panel corrected standard errors (PCSE) model was also deployed to confirm the robustness or reliability of the results (Table 4). PCSE is the best option along with the SYS GMM which address the cross-section dependency [124]. Therefore, the findings reveal the same results as described by system GMM.

**Table 4:** Robustness checking through PCSE.

	GDP Model	CO_2_ Model
**High Income Countries**		
Forward GVCs	0.124* (0.03)	−0.0792** (0.021)
Backward GVCs	−0.204**(0.07)	−0.0496**(0.013)
Foreign Direct Investment	−0.0056 (0.11)	−0.0132* (0.002)
Human Capital Index	0.791*(0.03)	1.021 (0.643)
Economic Freedom	0.0238*** (0.014)	−0.0604* (0.0054)
Constant	21.06 (0.076)	−4.9874 (0.7463)
R^2^	0.372	0.251
Wald chi^2^	10324.33* (0.000)	9657.22* (0.00)
**Upper Middle Income Countries**		
Forward GVCs	−0.396*(0.042)	3.056 (1.867)
Backward GVCs	0.512* (0.038)	−3.002* (0.41)
Foreign Direct Investment	−5.982 (3.847)	1.874 (0.603)
Human Capital Index	1.791* (0.107)	−5.962* (0.66)
Economic Freedom	0.0411* (0.004)	−0.1302***(0.074)
Constant	20.36 (0.033)	21.23 (0.122)
R^2^	0.258	0.273
Wald chi^2^	759.58* (0.000)	567.33* (0.00)
**Lower Middle Income Countries**		
Forward GVCs	0.0193 (0.147)	1.104* (0.037)
Backward GVCs	0.030* (0.006)	1.0072* (0.028)
Foreign Direct Investment	0.693* (0.08)	1.143* (0.044)
Human Capital Index	−0.362 (0.104)	0.4536 (0.892)
Economic Freedom	0.0233* (0.002)	−0.0482 (0.165)
Constant	22.17 (0.177)	−10.32 (0.546)
R^2^	0.293	0.305
Wald chi^2^	8954.33* (0.000)	4392.21* (0.00)

*, **, and *** denotes significance levels at 1 %, 5 %, and 10 % respectively.

## 5 Conclusion

Countries' participation in the GVC has attracted the attention of academicians, researchers, and policymakers around the globe due to its role in eco-friendly economic growth. The size of economies is one of the main determinants of countries' forward and backward GVC participation. Therefore, this study explored the role of F-GVC and B-GVC in eco-friendly economic growth (GDP and CO_2_ emissions). The SYS-GMM model was applied to control the endogeneity. The Fixed Effect (FE) model was used to check the robustness of the results.

This study presents some very fruitful insights regarding the role of F-GVC and B-GVC participation in economic growth and CO_2_ emissions in different economies. In the case of high-income economies, the F-GVC contributes to eco-friendly economic growth with a positive contribution to GDP and a reduction in CO_2_ emissions, while the B-GVC reduces both economic growth and CO_2_ emissions. In the case of upper-middle economies, the B-GVC contributes to eco-friendly economic growth with a positive contribution to GDP and a reduction in CO_2_ emissions. Thus, by increasing their B-GVC participation, upper-middle-income economies can experience eco-friendly economic growth. Both F-GVC and B-GVC are better for lower-middle income economies' economic growth, but they must contend with high CO_2_ emissions.

### 5.1 Policy implications

Based on study results, the following policy recommendations for different economies can be proposed: The high-income economies, which have well-established economic and environmental policies as well as sophisticated production methods, should also focus on B-GVC with F-GVC to enjoy more economic growth because it also lowers the emissions of CO_2_. For high-income economies, policymakers should focus on encouraging forward participation in Global Value Chains (F-GVCs) to boost GDP per capita. Emphasizing technological innovation, export-oriented industries, and attracting foreign direct investment in high value-added sectors can lead to economic growth. Additionally, measures to improve the efficiency of backward participation in GVCs (B-GVC) should be explored to mitigate any negative impacts on GDP per capita. Moreover, high-income countries can make backward participation in GVC more environmentally friendly and reduce CO_2_ emissions by implementing green procurement policies, facilitating technology transfer, enforcing stringent environmental standards, promoting circular economy principles, implementing sustainable supply chain management practices, and encouraging energy-efficiency measures in their backward linkages. By implementing these measures, high-income countries can significantly improve the environmental performance of backward participation in GVCs, leading to reduced CO_2_ emissions, and contributing to global efforts to mitigate climate change.

Upper-middle-income economies should focus on domestic value-added to make the contribution of F-GVC positive to economic growth. They should encourage R&D and technology upgrading for higher value-added goods, boost competitiveness in F-GVCs and economic growth, support export-oriented industries with incentives, and reduce trade barriers to enhance export performance. Moreover, they should promote diversification in F-GVC segments for economic resilience, reduce reliance on specific sectors, ensure the provision of incentives for sustainable practices, invest in education for a skilled workforce, and develop industrial clusters for collaboration and economies of scale. Upper-middle-income countries should incentivize forward-linked industries to adopt cleaner and more sustainable production techniques, invest in eco-friendly technologies to reduce emissions, promote the use of renewable energy, support research on sustainable solutions, and encourage eco-conscious product design.

Participation in both F-GVC and B-GVC is a great opportunity for lower-middle income economies that have very poor policies and limited resources with unsophisticated production methods to enjoy positive and higher economic growth and low CO_2_ emission. To achieve this, they should invest in green infrastructure to attract foreign direct investment and integrate better into GVCs. They can simplify trade and customs processes to encourage foreign investment and GVC participation. They should focus on education and skill programs to create a skilled workforce for GVC industries that can meet their demands of GVC industries. They should facilitate access to finance for SMEs to upgrade their production processes and effectively participate in GVCs. They can offer green incentives and subsidies to industries engaged in forward and backward participation, which adopt environmentally friendly practices. They can provide targeted incentives to attract investments in GVC-integrated sectors. They can encourage domestic and international collaboration to access knowledge and technology, which drives collective action and initiatives for greener GVC participation.

### 5.2 Limitations and future directions

The study provides the comprehensive understanding of crucial role of B-GVC and F-GVC participation in the growth of an economy as well as the CO_2_ emission across the different income nations. however, there are possible limitations that must be considered here. The current study could not focus the temporal scope means that technological transformation, changes in trade policies around the world and various economic event beyond the study period may have different impact on the relevance of the findings. Similarly, the current version of the study could not consider the sectoral differences among nations, that might impact the economic growth and CO_2_ emission.

The future studies must incorporate the sector-specific analysis, which may provide more robust outcomes. Similarly, the inclusion of other external factors such as stringency of environmental policies, green technologies and innovations may provide more fruitful insights for developing effective policy recommendations. Moreover, the studies may perform the comparative analysis based on the different policy framework to foster the GVC participation while lowering the CO_2_ emission.

## Funding details

The authors declare that no funds, grants, or other support were received during the preparation of this manuscript.

## Data availability statement

Data will be made available on request.

## CRediT authorship contribution statement

**Amar Razzaq:** Writing – review & editing, Writing – original draft, Visualization, Supervision, Project administration, Methodology, Funding acquisition, Formal analysis, Conceptualization. **Pomi Shahbaz:** Writing – review & editing, Methodology, Investigation, Formal analysis, Data curation, Conceptualization. **Shamsheer ul Haq:** Writing – review & editing, Validation, Methodology, Formal analysis, Data curation, Conceptualization. **Yewang Zhou:** Writing – review & editing, Visualization, Methodology, Investigation, Funding acquisition. **Sahar Erfanian:** Writing – review & editing, Validation, Software. **Azhar Abbas:** Writing – review & editing, Validation, Software, Investigation.

## Declaration of competing interest

The authors declare that they have no known competing financial interests or personal relationships that could have appeared to influence the work reported in this paper.

## Figures and Tables

**Table:** 

Lower-middle income countries	Middle-upper income countries	High-income countries
Algeria, Angola, Bangladesh, Belize, Benin, Bolivia, Cambodia, Cameroon, Cote dIvoire, Djibouti, Egypt, El Salvador, Ghana, Haiti, Honduras, India, Indonesia, Kenya, Kyrgyzstan, Laos, Lesotho, Mauritania, Mongolia, Morocco, Myanmar, Nepal, Nicaragua, Nigeria, Pakistan, Papua New Guinea, Philippines, Samoa, Senegal, Sri Lanka, Tajikistan, Tunisia, Ukraine, Tanzania, Uzbekistan, Vanuatu, Venezuela, Viet Nam	Albania, Argentina, Armenia, Azerbaijan, Belarus, Bosnia and Herzegovina, Botswana, Brazil, Bulgaria, China, Colombia, Costa Rica, Cuba, Dominican Republic, Ecuador, Fiji, Gabon, Georgia, Guatemala, Jamaica, Jordan, Kazakhstan, Lebanon, Malaysia, Maldives, Mauritius, Mexico, Namibia, Panama, Paraguay, Peru, Romania, Russia, South Africa, Thailand, Turkey	Austria, Australia, Bahamas, Bahrain, Barbados, Belgium, Brunei, Canada, Chile, Croatia, Cyprus, Czech Republic, North Korea, Denmark, Estonia, Finland, France, Germany, Greece, Hong Kong, Hungary, Iceland, Ireland, Israel, Italy, Japan, Kuwait, Latvia, Lithuania, Luxembourg, Malta, Netherlands, New Zealand, Norway, Oman, Poland, Portugal, Saudi Arabia, Singapore, Slovakia, Slovenia, Spain, Sweden, Switzerland, Trinidad and Tobago, UK, USA, Uruguay
